# A saúde indígena na atenção especializada: perspectiva dos profissionais de saúde em um hospital de referência no Mato Grosso do Sul, Brasil

**DOI:** 10.1590/0102-311XPT094622

**Published:** 2024-07-29

**Authors:** Fabiana Casagranda, Verônica Gronau Luz, Catia Paranhos Martins, Raquel Paiva Dias-Scopel, Ricardo Fernandes, Wanaline Fonseca

**Affiliations:** 1 Universidade Federal da Grande Dourados, Dourados, Brasil.; 2 Instituto Leônidas & Maria Deane, Fundação Oswaldo Cruz, Manaus, Brasil.; 3 Empresa Brasileira de Serviços Hospitalares, Dourados, Brasil.

**Keywords:** Saúde de Populações Indígenas, Serviços de Saúde do Indígena, Sistemas Locais de Saúde, Assistência Hospitalar, Pessoal de Saúde, Health of Indigenous Peoples, Indigenous Health Services, Local Health Systems, Hospital Care, Health Personnel, Salud de Poblaciones Indígenas, Servicios de Salud del Indígena, Sistemas Locales de Salud, Atención Hospitalaria, Personal de Salud

## Abstract

Trata-se de estudo transversal com o objetivo de identificar a perspectiva dos profissionais de saúde e residentes acerca dos desafios enfrentados no atendimento aos usuários indígenas em um hospital referência no Mato Grosso do Sul, estado brasileiro que comporta a segunda maior população indígena do país. O estudo utilizou questionário semiestruturado *online*, enviado por *e-mail* a cada trabalhador(a) no período entre junho e agosto de 2020. As variáveis discretas foram sintetizadas em média e desvio padrão e mediana e intervalo interquartil (nível de significância de 5%). Participaram do estudo 230 profissionais de saúde e 29 residentes. Entre eles, apenas 14,7% conheciam as etnias atendidas, e 60,2% nunca havia presenciado práticas tradicionais no hospital, indicando baixa articulação entre as formas de cuidado biomédico e indígena. Ao confrontar respostas dos residentes e profissionais, observou-se que residentes têm uma visão mais positiva acerca da aproximação com o contexto indígena, sugerindo que consideram importante melhorar essa articulação. Na comparação entre categorias profissionais, destacam-se algumas divergências de opiniões da categoria médica em relação à assistência. Além disso, profissionais e residentes demonstraram sentir algum nível de dificuldade no atendimento à população indígena. Os resultados evidenciam a centralidade do modelo biomédico, o desconhecimento dos profissionais sobre o contexto das comunidades atendidas e a desvalorização de suas práticas. Além disso, contribuem para discussões sobre as políticas de saúde nos diversos níveis de atenção e gestão, bem como na qualificação da assistência hospitalar aos indígenas.

## Abstract (en)

This cross-sectional study aims to identify the perspective of healthcare professionals and residents regarding the challenges faced in providing care to Indigenous users in a reference hospital in Mato Grosso do Sul, a Brazilian state with the second-largest Indigenous population in the country. The study used a semi-structured online questionnaire emailed to each worker between June and August 2020. The discrete variables were summarized as mean and standard deviation and median and interquartile range (5% significance level). Two hundred thirty healthcare professionals and 29 residents participated in the study. Among the findings, only 14.7% of participants knew the ethnicities served, and 60.2% had never witnessed traditional practices in the hospital, indicating low articulation between biomedical and Indigenous forms of care. When comparing responses from residents and professionals, residents were noted to have a more positive view of approaching the Indigenous context, suggesting that they consider it essential to improve this articulation. When comparing professional categories, some differences of opinion among the medical category stand out concerning assistance. In addition, professionals and residents demonstrated some level of difficulty in caring for the Indigenous population. The results highlight the centrality of the biomedical model, the professionals’ lack of knowledge about the context of the communities served, and the devaluation of their practices. The findings contribute to discussions about healthcare policies at different levels of care and management and the qualification of hospital care for Indigenous people.

## Resumen

Se trata de un estudio transversal con el objetivo de identificar la perspectiva de los profesionales de la salud y los médicos residentes sobre los desafíos que enfrentan en la atención a los usuarios indígenas en un hospital de referencia en Mato Grosso del Sur, el estado brasileño que tiene la segunda población indígena más grande del país. El estudio utilizó un cuestionario semiestructurado en línea, enviado por correo electrónico para cada trabajador y trabajadora entre los meses de junio y agosto de 2020. Se sintetizaron las variables discretas en media y desviación estándar, y mediana y rango intercuartílico (nivel de significación del 5%). Participaron del estudio 230 profesionales de la salud y 29 médicos residentes. En los resultados, solo el 14,7% de los participantes conocían las etnias atendidas, y el 60,2% nunca había presenciado prácticas tradicionales en el hospital, lo que indica una baja articulación entre las formas de atención biomédica e indígena. Al confrontar respuestas de los médicos residentes y los profesionales, se constató que los médicos residentes tienen una perspectiva más positiva sobre el acercamiento al contexto indígena, lo que sugiere que ellos consideran importante mejorar esta articulación. Al comparar las categorías profesionales, se destacan algunas divergencias de opinión de la categoría médica en relación con la asistencia. Además, los profesionales y los médicos residentes demostraron un cierto nivel de dificultad en la atención a la población indígena. Los resultados demuestran la centralidad del modelo biomédico, la falta de conocimiento de los profesionales sobre el contexto de las comunidades atendidas y la desvalorización de sus prácticas. Los resultados contribuyen a las discusiones sobre las políticas de salud en varios niveles de atención y gestión, así como en la calificación de la atención hospitalaria a los indígenas.

## Introdução

No Brasil, desde 1999, o Subsistema de Atenção à Saúde Indígena (Sasi), criado no âmbito do Sistema Único de Saúde (SUS), organiza e estrutura os serviços de saúde direcionados aos povos indígenas por meio de 34 Distritos Sanitários Especiais Indígenas (DSEI), almejando considerar as especificidades geográficas e organizações sociais dos 305 diferentes povos ^1^ ^,^ ^2^.

Em 2002, foi instituída a Política Nacional de Atenção à Saúde dos Povos Indígenas (PNASPI) que apresentou diretrizes sobre a assistência a pacientes indígenas nos serviços do SUS, reconhecendo as especificidades culturais, na tentativa de articular as diferentes formas de atenção, incluindo a biomedicina e os saberes considerados “tradicionais” ^3^. O SasiSUS é responsável pela atenção primária à saúde (APS) e, quando há necessidade de atendimento especializado, os pacientes indígenas são encaminhados aos serviços de referência.

Os povos indígenas enfrentam diversos desafios no acesso e na utilização dos serviços de saúde, tanto na APS como na atenção especializada. Apesar de algumas diferenças entre os níveis de atenção, os principais obstáculos descritos na literatura são: limitações geográficas, organizacionais e culturais; ausência de intérpretes; falta de capacitação dos profissionais para lidar com diferentes etnias; além de racismo e discriminação vivenciados pelos povos indígenas durante o atendimento ^4^ ^,^ ^5^ ^,^ ^6^.

As populações indígenas apresentam os piores indicadores de saúde em comparação às populações não indígenas no Brasil. As razões dessas disparidades são complexas e se devem, em parte, ao tratamento histórico dos povos originários desde a colonização, enfrentando negações de direitos humanos básicos há mais de 500 anos ^7^ ^,^ ^8^. Poucos estudos têm explorado como os profissionais percebem o acesso e o atendimento ofertado aos indígenas nos serviços de saúde ^5^ ^,^ ^9^ ^,^ ^10^. Os dados são ainda mais escassos em relação à atenção de média e alta complexidade ^5^ ^,^ ^11^ ^,^ ^12^ ^,^ ^13^.

Nesse sentido, este estudo visa discutir perspectivas e desafios enfrentados por profissionais de saúde e residentes do Hospital Universitário da Universidade Federal da Grande Dourados (HU-UFGD), referência no Mato Grosso do Sul quanto ao atendimento à população indígena. Tais reflexões podem amparar a avaliação e a atualização de políticas, ações e processos de educação permanentes em saúde, que considerem o enfrentamento das iniquidades étnico-raciais.

## Métodos

Trata-se de um estudo transversal desenvolvido no HU-UFGD em Dourados, Mato Grosso do Sul. O hospital presta assistência aos 34 dos 79 municípios do estado. A região é composta por 6 dos 14 polos base do DSEI de Mato Grosso do Sul, incluindo o de Dourados, que é o maior do estado e do Brasil em quantitativo de pessoas atendidas.

O Mato Grosso do Sul tem a segunda maior população indígena do Brasil, somando 83.241 pessoas, sendo mais prevalentes na região os Guarani Kaiowá, Guarani Ñandeva e os Terena. O atendimento a indígenas no HU-UFGD corresponde a aproximadamente 10,9% do total de internações hospitalares ^14^ ^,^ ^15^.

Em 2018, o hospital implantou o Núcleo de Saúde Indígena (NSI) com o objetivo de promover um cuidado mais adequado e implementar o recurso do Incentivo à Atenção Especializada aos Povos Indígenas (IAE-PI), regulamentado na *Portaria nº 2.663/2017* ^16^. O recurso foi recebido em 2019 e tem como finalidade qualificar a atenção especializada no SUS, garantindo a complementaridade da atenção aos povos indígenas.

Foram convidados para a pesquisa todos os trabalhadores da saúde da linha materno-infantil do HU-UFGD que prestam assistência a usuários indígenas. Também foram convidados a participar todos os pós-graduandos das Residências Médicas, multi e uniprofissionais, que realizavam assistência nessa linha de cuidado. Optou-se pela participação de profissionais da linha materno-infantil uma vez que atende a maioria das pessoas indígenas.

Os setores que compõem a linha são: Clínica Pediátrica, Unidade de Terapia Intensiva (UTI) Neonatal, Unidade de Cuidados Intermediários (UCI) Neonatal, UTI Pediátrica, Centro Obstétrico, Alojamento Conjunto (maternidade), Pronto Atendimento Ginecológico/Obstétrico e Casa da Gestante, Bebê e Puérpera.

A linha materno-infantil tinha, até 2020, 324 profissionais atuantes na assistência, sendo: 49 médicos, 65 enfermeiros, 179 técnicos de enfermagem, 20 fisioterapeutas, 3 nutricionistas, 3 psicólogas, 2 assistentes sociais, 2 fonoaudiólogos, 1 farmacêutico e 1 terapeuta ocupacional. Devido à pandemia causada pela COVID-19, alguns profissionais foram realocados entre 2020 e 2021.

Os participantes dos programas de Residência que atuam na linha materno-infantil são: os residentes multiprofissionais, sendo 11 com ênfase em atenção cardiovascular e 11 em atenção à saúde indígena; 8 residentes multiprofissionais em saúde materno-infantil; 6 residentes uniprofissionais em enfermagem obstétrica; além de 24 residentes médicos, sendo 13 em pediatria e 11 em ginecologia e obstetrícia, totalizando 60 residentes. Foram excluídos da pesquisa os profissionais de outros setores e os residentes que ainda não atuaram na linha materno-infantil.

A coleta de dados foi realizada mediante o envio por e-mail de um questionário do Google Forms (https://forms.google.com). Os indivíduos que aceitaram responder digitalmente assinaram o Termo de Consentimento Livre e Esclarecido. O questionário era semiestruturado, com questões socioeconômicas e demográficas. Para os residentes, também perguntou-se sobre formação básica, qual programa de Residência estavam inseridos e ano do curso. Questões sobre as etnias locais e práticas de cuidado dentro do hospital, além da percepção do atendimento aos usuários indígenas foram feitas a todos os profissionais, bem como sobre a articulação da medicina hospitalar com os cuidados tradicionais. Foram também questionados sobre a atuação em equipe de trabalho, discussão de casos e relacionamento interpessoal, a articulação com outros pontos da rede de atenção e educação permanente. O questionário foi adaptado da proposta *Diagnóstico Compartilhado na Gestão da Atenção Hospitalar* de Pinto ^17^.

As questões eram de múltipla escolha ou com indicadores em escala de 1 a 10, sendo 1 “pouco importante” ou “nunca” e 10 “muito importante” ou “sempre”. Para melhor compreensão de alguns resultados, as questões foram divididas em três blocos: percepções pessoais, percepções sobre a articulação com cuidado tradicional e aproximação com as vivências da população indígena; percepções sobre o trabalho em equipe na assistência ao usuário indígena; e percepções sobre a articulação da equipe de saúde hospitalar com a rede de cuidado em saúde indígena.

Foram enviados três e-mails para cada profissional, com intervalo médio de dez dias para cada tentativa. O questionário foi enviado para os e-mails institucional e pessoal dos trabalhadores e residentes. Todos os setores foram avisados previamente da pesquisa e houve divulgação pessoalmente e em grupos de WhatsApp. Os e-mails foram fornecidos pela instituição. Os questionários foram enviados nos meses de agosto e setembro de 2020 e podiam ser respondidos uma única vez.

Os dados foram organizados e registrados em banco de dados no programa Microsoft Office Excel 365 (https://products.office.com/). A análise estatística foi realizada no programa estatístico Stata, versão 13.1 para Windows (https://www.stata.com).

As variáveis discretas foram sintetizadas em média e desvio padrão e mediana e intervalo interquartil. Já as categóricas foram descritas em categorias e frequência a partir do aparecimento nos grupos estabelecidos. Para avaliar a distribuição dos dados nas variáveis discretas, foi aplicado o teste de normalidade de Shapiro-Wilk. Tanto no caso de não normalidade quanto em situações de heterogeneidade das variâncias, foram utilizados testes não-paramétricos. Nas comparações entre dois grupos, foram utilizados o teste t de Student ou o teste de Mann-Whitney. Nas comparações entre mais de dois grupos, foi utilizado o teste de Kruskal-Wallis. Em caso de diferenças estatisticamente significativas, o teste de Dunn com correção para múltiplos testes em pares (método de Bonferroni) foi aplicado. Para todos os testes, foi adotado o nível de significância de 5%.

Esta pesquisa foi aprovada pela Comissão de Avaliação de Pesquisa e Extensão do HU-UFGD (parecer nº 101.2019) e pelo Comitê de Ética em Pesquisa da UFGD (parecer nº 4.050.973).

## Resultados

Dos 324 profissionais de saúde atuantes na linha materno-infantil do HU-UFGD, 230 (71%) participaram da pesquisa.

A maioria dos profissionais era mulher (83,5%), sendo metade deles com pós-graduação completa (49,1%). Os profissionais da área de enfermagem somaram 79,6% de todos os respondentes, seguido dos médicos (10,8%), fisioterapeutas (5,2%) e demais profissionais (4,4%), como mostra a Tabela 1.

**Tabela 1:** Perfil sociodemográfico dos servidores da linha materno-infantil do Hospital Universitário da Universidade Federal da Grande Dourados (HU-UFGD). Dourados, Mato Grosso do Sul, Brasil, 2020 (n = 230).

Variáveis	n	%
Sexo		
Feminino	192	83,5
Masculino	38	16,5
Escolaridade		
Curso técnico	64	27,8
Graduação completa	32	13,9
Pós-graduação *lato sensu* (completa)	113	49,1
Mestrado completo	19	8,3
Doutorado completo	2	0,9
Profissão/Função		
Técnico(as) de enfermagem	123	53,5
Enfermeiro(a)	60	26,1
Médico(a)	25	10,8
Fisioterapeuta	12	5,2
Psicólogo(a)	2	0,9
Nutricionista	2	0,9
Fonoaudiólogo(a)	2	0,9
Terapeuta ocupacional	2	0,9
Farmacêutico(a)	1	0,4
Assistente social	1	0,4
Tempo de serviço no HU-UFGD * (anos)		
< 5	46	20,4
5-10	125	55,1
10-15	46	20,4
≥ 15	9	3,9
Local de lotação **		
Alojamento Conjunto	31	13,5
Casa da Gestante, Bebê e Puérpera	6	2,6
Centro Obstétrico	19	8,3
Clínica Pediátrica	56	24,5
Pronto Atendimento Ginecológico/Obstétrico	14	6,1
UTI Neonatal	28	12,2
UCI Neonatal	41	17,9
UTI Pediátrica	34	14,9
Naturalidade (região de nascimento)		
Norte	5	4,6
Nordeste	23	10,0
Centro-oeste	136	59,1
Sudeste	33	14,3
Sul	33	14,3

UCI: unidade de cuidados intermediários; UTI: unidade de terapia intensiva.

* n = 226;

** n = 229.

Em relação ao tempo de serviço no HU-UFGD, 75,5% dos trabalhadores tinham entre 5 e 15 anos e quase 60% eram naturais do Centro-oeste, sendo 94,8% desses do Mato Grosso do Sul.

Do total de 60 residentes convidados a participarem da pesquisa, 29 (48,3%) responderam ao questionário. O perfil sociodemográfico dos participantes encontra-se na Tabela 2.

**Tabela 2:** Perfil sociodemográfico dos residentes que atuam na linha materno-infantil do Hospital Universitário da Universidade Federal da Grande Dourados (HU-UFGD). Dourados, Mato Grosso do Sul, Brasil, 2020 (n = 29).

Variáveis	n	%
Sexo		
Feminino	25	86,2
Masculino	4	13,8
Escolaridade		
Graduação completa	23	79,4
Pós-graduação *lato sensu* (completa)	3	10,3
Mestrado completo	3	10,3
Residência que está cursando *		
Residência Médica em Pediatria	1	3,7
Residência Médica em Ginecologia e Obstetrícia	0	0,0
Residência Uniprofissional em Enfermagem Obstétrica	5	18,5
Residência Multiprofissional: Materno-Infantil	9	33,3
Residência Multiprofissional: Atenção Cardiovascular	5	18,5
Residência Multiprofissional: Atenção à Saúde Indígena	7	26,0
Formação profissional		
Medicina	3	10,3
Enfermagem	8	27,6
Fisioterapia	1	3,5
Nutrição	8	27,6
Psicologia	9	31,0
Ano de residência *		
1º	7	35,0
2º	20	65,0
Naturalidade (região de nascimento)		
Norte	1	3,4
Nordeste	1	3,4
Centro-oeste	16	55,1
Sudeste	4	13,7
Sul	7	24,1

* n = 27.

Percebe-se que a maioria dos residentes também são do sexo feminino, sendo que 20,6% já tinham alguma pós-graduação completa (*lato* ou *strictu sensu*). Entre esses, as categorias que mais colaboraram no estudo foram psicologia (nove), enfermagem (oito) e nutrição (oito). Em relação aos programas, os residentes que mais participaram foram os da Residência Multiprofissional em Saúde Materno-infantil, e houve baixa participação dos programas de Residência Médica. Do total de 24 residentes médicos, apenas três participaram do estudo (12,5%). Nenhum interlocutor da pesquisa era indígena.

A Tabela 3 mostra os dados sobre o conhecimento relacionado a etnias e cuidados tradicionais pelos trabalhadores e residentes que atuam no HU-UFGD. Apenas um dos participantes respondeu não ter atendido usuários indígenas no hospital.

**Tabela 3:** Conhecimento do número de etnias e cuidados tradicionais indígenas pelos profissionais e residentes que atuam na linha materno-infantil do Hospital Universitário da Universidade Federal da Grande Dourados (HU-UFGD). Dourados, Mato Grosso do Sul, Brasil, 2020.

Variáveis	Total	Profissionais	Residentes
	n (%)	n (%)	n (%)
Atende usuários indígenas			
Sim	258 (99,6)	229 (99,6)	29 (100)
Não	1 (0,4)	1 (0,4)	0 (0,0)
Sabe quantas etnias indígenas existem em Dourados			
Sim	106 (40,9)	91 (39,6)	15 (51,7)
Não	153 (59,1)	139 (60,4)	14 (48,3)
Se sim, quantas etnias			
1	3 (2,5)	3 (2,9)	0 (0,0)
2	48 (40,7)	41 (40,1)	7 (43,7)
3	47 (39,9)	40 (39,2)	7 (43,7)
4	16 (13,5)	14 (13,7)	2 (12,5)
5 ou mais	4 (3,4)	4 (3,9)	0 (0,0)
Já presenciou algum cuidado tradicional			
Sim	98 (37,8)	91 (39,5)	7 (24,1)
Não	156 (60,2)	134 (58,3)	22 (75,9)
Não sabe/Não respondeu	5 (2,0)	5 (2,2)	0 (0,0)
Se sim, qual(is) cuidado(s)			
Apenas reza/canto	52 (53,1)	50 (54,9)	2 (28,6)
Reza/canto + medicamentos	15 (15,3)	15 (16,5)	0 (0,0)
Reza/canto + medicamentos + outros	1 (1,0)	1 (1,1)	0 (0,0)
Reza/canto + outros (sem medicamentos)	3 (3,1)	3 (3,3)	0 (0,0)
Apenas medicamentos tradicionais	12 (12,2)	7 (7,7)	5 (71,4)
Outros	15 (15,3)	15 (16,5)	0 (0,0)

Quando perguntados a respeito das etnias existentes em Dourados, a maioria dos profissionais respondeu não ter esse conhecimento (60,4%). Já com os residentes, pouco mais da metade afirmou saber (51,7%).

Do total de 106 profissionais e residentes que afirmaram conhecer as etnias, apenas 47 (39,9%) souberam responder corretamente as mais predominantes em Dourados - Guarani Ñandeva, Guarani Kaiowá e Terena - e desses, sete eram da Residência Multiprofissional (Tabela 3), ou seja, dos 259 profissionais efetivos e residentes que participaram, apenas 14,7% conheciam as etnias que atendiam (porcentagem não apresentada na Tabela 3).

A maioria dos profissionais e dos residentes não havia presenciado, até o momento da pesquisa, nenhum cuidado tradicional no hospital (60,2%), e, entre aqueles que já haviam presenciado, o mais prevalente foi “apenas reza/canto” (53,1%).

Os resultados da Tabela 4 são referentes às perguntas sobre o atendimento aos usuários indígenas, e a comparação da variabilidade das respostas (1 a 10) referentes às questões sobre a assistência a indígenas realizada por trabalhadores e residentes do hospital. Os resultados mostraram que existe diferença estatisticamente significativa (p = 0,010) entre a percepção de trabalhadores e residentes para a dificuldade na assistência aos indígenas, tendo residentes um pouco menos de dificuldade (média de 5,11) que trabalhadores (média de 6,08). Os residentes tiveram pontuações mais altas (mediana de 10) sobre considerar adequada que ocorra a articulação da medicina hospitalar e o cuidado tradicional do que os trabalhadores (mediana de 7). Também houve diferença significativa (p < 0,001) em relação à importância de se aproximar do contexto indígena local, para melhorar a assistência, sendo que os residentes tiveram uma percepção de maior importância (mediana de 10) para essa aproximação.

**Tabela 4:** Medidas de posição e variabilidade das respostas (1 a 10) referentes às questões sobre a assistência aos usuários indígenas realizadas por trabalhadores e residentes do Hospital Universitário da Universidade Federal da Grande Dourados (HU-UFGD). Dourados, Mato Grosso do Sul, Brasil, 2020.

Questões	Trabalhadores		Residentes		Valor de p
	n	Média ± DP	n	Média ± DP	
Percepções pessoais, articulação com a medicina tradicional e aproximação com a realidade					
Na assistência aos usuários indígenas, o quanto você sente dificuldade?	220	6,08 ± 1,87	28	5,11 ± 1,95	0,010 *
Considera adequado realizar a articulação da medicina hospitalar e o cuidado tradicional indígena?	191	6,70 ± 2,50	27	8,70 ± 2,43	< 0,001 *
Considera importante, para melhorar a sua assistência a esses povos, aproximar-se mais do contexto indígena para além do ambiente hospitalar?	211	8,57 ± 1,91	28	9,82 ± 0,55	< 0,001 **
Percepções sobre o trabalho em equipe na assistência a usuários(as) indígenas					
A equipe de trabalho consegue atuar de maneira harmonizada?	215	7,66 ± 2,03	28	6,10 ± 2,23	< 0,001 *
Os casos dos usuários indígenas são discutidos em equipe?	203	5,50 ± 2,88	27	5,33 ± 2,62	0,581 *
A equipe aborda os fatores subjetivos dos usuários indígenas?	203	5,46 ± 2,63	28	5,14 ± 2,76	0,545 **
A equipe aborda os fatores sociais e étnicos?	195	5,09 ± 2,68	28	4,96 ± 2,92	0,892 **
A equipe realiza atividades de educação com os usuários indígenas e seus familiares para autocuidado?	203	6,59 ± 2,70	29	5,62 ± 2,70	0,071 *
A equipe discute casos, troca de informações e opiniões aproveitando-se dos diversos nos cuidados aos usuários indígenas?	197	5,53 ± 2,73	27	5,07 ± 2,93	0,376 **
Percepções sobre a articulação da equipe hospitalar com a rede de saúde indígena					
A equipe do hospital comunica a alta do usuário à rede de saúde indígena?	184	8,21 ± 2,35	25	6,84 ± 2,66	0,008 *
Em situações mais complexas, a equipe do hospital realiza a discussão do caso com as equipes de saúde indígena da rede?	174	6,92 ± 2,83	21	5,95 ± 3,01	0,126 **

DP: desvio padrão.

Nota: os valores de p em negrito correspondem às diferenças estatisticamente significativas (p < 0,05).

* Teste t de Student independente;

** Teste de Mann-Whitney

Os residentes consideram que a atuação da equipe é menos combinada/harmonizada (mediana de 6,5) com os demais profissionais, em relação à percepção dos trabalhadores (mediana de 8) (p < 0,001). Ainda, foi considerada significativa (p = 0,008) a diferença de percepção entre os profissionais e residentes quanto à comunicação da alta hospitalar do usuário à Secretaria de Saúde Indígena (SESAI), ao NSI ou à Casa de Saúde Indígena (CASAI); para essa questão os trabalhadores tiveram uma percepção mais positiva (mediana de 9).

Não foi encontrada diferença significativa entre as percepções de profissionais e residentes para as perguntas relacionadas à atuação em equipe, como: discussão de casos de pacientes indígenas; abordagem da equipe em relação a fatores subjetivos, étnicos e sociais dos usuários indígenas; e realização de atividades de educação com usuários e familiares.

A Tabela 5 mostra a variabilidade das respostas (de 1 a 10) nas mesmas questões sobre a assistência aos usuários indígenas, comparando-as por categoria profissional: médicos, enfermeiros, técnicos de enfermagem e demais profissionais (fisioterapeutas, nutricionistas, psicólogos, fonoaudiólogos, terapeutas ocupacionais, farmacêuticos e assistentes sociais). Sobre as percepções na dificuldade da assistência, não houve diferenças estatisticamente significativas entre as profissões (p = 0,417). Apesar disso, para essa questão, verificou-se que as categorias enfermeiros e técnicos de enfermagem tiveram as menores médias (6), quando comparadas às demais categorias, demonstrando que se sentem mais seguras no atendimento a esses povos.

**Tabela 5:** Medidas de posição e variabilidade das respostas (1 a 10) às questões referente à assistência aos usuários indígenas realizadas pelos servidores do Hospital Universitário da Universidade Federal da Grande Dourados (HU-UFGD). Dourados, Mato Grosso do Sul, Brasil, 2020.

Questões	Médicos		Enfermeiros		Técnicos de enfermagem		Demais profissionais		Valor de p
	n	Média ± DP	n	Média ± DP	n	Média ± DP	n	Média ± DP	
Percepções pessoais, articulação com a medicina tradicional e aproximação com a realidade									
Na assistência aos usuários indígenas, quanto você sente dificuldade?	24	6,79 ± 1,72 ^a^	59	6,05 ± 1,71 ^a^	118	5,95 ± 1,94 ^a^	19	6,11 ± 2,05 ^a^	0,417
Considera adequado realizar a articulação da medicina hospitalar e o cuidado tradicional indígena?	23	7,48 ± 2,00 ^a^	55	7,27 ± 2,16 ^a^	95	6,21 ± 2,68 ^a^	18	6,56 ± 2,59 ^a^	0,058
Considera importante se aproximar do contexto indígena para além do ambiente hospitalar?	22	8,27 ± 2,31 ^a,b^	57	9,04 ± 1,38 ^a^	112	8,25 ± 2,09 ^a^	20	9,35 ± 1,09 ^b^	0,021
Percepções sobre o trabalho em equipe na assistência a usuários(as) indígenas									
A equipe de trabalho consegue atuar de maneira harmonizada?	24	8,50 ± 1,91 ^a^	58	7,34 ± 2,09 ^a^	114	7,65 ± 2,00 ^a^	19	7,58 ± 1,98 ^a^	0,060
Os casos dos usuários indígenas são discutidos em equipe?	22	7,18 ± 3,17 ^a^	58	5,41 ± 2,66 ^b^	105	5,06 ± 2,91 ^b^	18	6,28 ± 2,19 ^a,b^	0,003
A equipe aborda os fatores subjetivos dos usuários indígenas?	21	7,09 ± 2,81 ^a^	59	5,53 ± 2,35 ^b^	109	5,15 ± 2,68 ^b^	13	5,00 ± 2,35 ^b^	0,008
A equipe aborda os fatores sociais e étnicos?	22	6,86 ± 2,89 ^a^	59	5,05 ± 2,32 ^b^	100	4,77 ± 2,76 ^b^	15	4,93 ± 2,43 ^a,b^	0,022
A equipe realiza atividades de educação com os usuários indígenas e seus familiares para autocuidado?	22	8,64 ± 1,05 ^a^	58	6,47 ± 2,49 ^b^	108	6,28 ± 2,90 ^b^	15	6,33 ± 2,53 ^b^	0,001
A equipe discute casos, troca de informações e opiniões aproveitando-se dos diversos nos cuidados aos usuários indígenas?	22	7,59 ± 2,24 ^a^	57	4,89 ± 2,38 ^b^	100	5,30 ± 2,89 ^b^	18	6,28 ± 2,11 ^a,b^	< 0,001
Percepções sobre a articulação da equipe de saúde hospitalar com a rede de saúde indígena									
A equipe do hospital comunica a alta do usuário à rede de saúde indígena?	21	8,33 ± 2,27 ^a^	48	7,60 ± 2,81 ^a^	101	8,49 ± 2,04 ^a^	14	8,14 ± 2,68 ^a^	0,183
Em situações mais complexas e de maior vulnerabilidade a equipe do hospital realiza a discussão do caso com as equipes de saúde indígena da rede?	20	8,00 ± 2,70 ^a^	53	6,32 ± 3,11 ^a^	86	7,09 ± 2,60 ^a^	15	6,60 ± 2,97 ^a^	0,083

DP: desvio padrão.

Nota: os valores de p em negrito correspondem às diferenças estatisticamente significativas (p < 0,05). Letras diferentes (a,b) nas linhas indicam diferença estatisticamente significativa pelo teste de Dunn com correção para múltiplos testes em pares pelo método de Bonferroni. Letras iguais nas linhas indicam que não há diferença estatisticamente significativa.

* Teste de Kruskal-Wallis.

Para considerar adequado realizar articulação com a medicina tradicional, não houve diferenças significativas entre as percepções das diferentes categorias profissionais, ou seja, a maioria percebe como positiva essa articulação dentro do hospital. Sobre a importância de se aproximar do contexto indígena fora do ambiente hospitalar, a categoria dos enfermeiros e dos técnicos de enfermagem não diferiram significativamente dos médicos, embora tenham diferido significativamente das demais categorias profissionais (p = 0,021). Para o restante das comparações, não houve diferenças significativas, mostrando uma percepção parecida entre as categorias profissionais.

Sobre o trabalho em equipe na assistência aos usuários indígenas, a questão sobre a atuação da equipe de maneira harmonizada/combinada foi similar entre as categorias profissionais (mediana entre 8 e 9). Houve diferença estatisticamente significativa (p = 0,003) sobre a discussão de casos em equipe, já que médicos atribuíram maior pontuação (mediana de 9) para essa pergunta, enquanto enfermeiros e técnicos tiveram percepções mais negativas (mediana de 5).

Outra diferença significativa (p = 0,008) foi em relação à equipe abordar os fatores subjetivos dos usuários indígenas. Os médicos foram a categoria que mais considerou fazer essa abordagem (mediana de 8), enquanto as demais categorias não diferiram entre si (mediana entre 5 e 6) nesse olhar.

Em relação à equipe abordar os fatores sociais e étnicos, e sobre as trocas de informações e opiniões em equipe, houve diferenças (p = 0,022) entre a categoria médica (mediana de 8), que diferiu dos enfermeiros e técnicos de enfermagem (mediana de 5), mas não dos demais profissionais. Essa diferença indica que médicos têm uma percepção distinta da abordagem da equipe sobre o usuário indígena e que consideram realizar a abordagem de fatores sociais e étnicos. Sobre a equipe realizar atividades de educação com usuários indígenas para o autocuidado, novamente, houve uma melhor percepção da categoria médica (mediana de 9), que diferiu estatisticamente das demais categorias (mediana de 7) (p = 0,001).

Portanto, percebe-se que, mesmo quando não houve diferenças significativas, a média e a mediana da categoria médica são, na maioria das questões, maiores que as dos outros grupos, tendo percepções mais positivas em relação ao atendimento a usuários indígenas e relacionamento com a equipe. Os enfermeiros e técnicos de enfermagem variaram quanto às percepções do atendimento, tendo percepções ora mais positivas, ora menos. No entanto, foram essas duas categorias com as piores percepções sobre a assistência aos indígenas.

## Discussão

Em relação às profissões participantes, mais da metade dos profissionais é composta por técnicos de enfermagem, seguida por enfermeiros e médicos. No entanto, proporcionalmente, a enfermagem foi a profissão que mais colaborou com o estudo (92,3%), seguido pelos técnicos de enfermagem (68,7%), do total de profissionais incluídos na pesquisa. Já a medicina foi, entre todas, a profissão com menor engajamento (51%). Em relação aos residentes também foi baixa a participação de médicos.

Quanto à baixa participação da categoria médica (profissionais e residentes médicos), pode-se argumentar que o estudo foi realizado em meio à pandemia de COVID-19 e os esforços dessa categoria estavam direcionados ao tratamento de uma doença desconhecida. Entretanto, a rotina da enfermagem e demais profissionais também sofreram alterações. O modelo biomédico tende a localizar a medicina como a responsável pela cura e a enfermagem pelos cuidados ^18^ e a ignorar as outras formas de atenção à saúde e doença ^19^.

Múltiplos fatores podem ter influenciado a menor adesão à pesquisa dos residentes multiprofissionais em relação aos profissionais do hospital. A divulgação presencial da pesquisa ocorreu durante a pandemia de COVID-19, que gerou alterações na dinâmica do hospital. Por uma decisão autocrática da direção, referendada pela intervenção na universidade, os residentes não médicos foram impedidos de seguirem com suas formações no hospital. Tais decisões geraram fragilidades e medos nos residentes multiprofissionais, o que pode ter provocado diferença na adesão à pesquisa. Outro fator da baixa participação desses interlocutores é a sobrecarga de trabalho e alta carga horária das residências (60 horas semanais).

### Etnias e etnocentrismo

Considerando o contingente populacional indígena do Mato Grosso do Sul, o atendimento hospitalar a usuários indígenas no HU-UFGD é uma demanda rotineira dos profissionais. Somente no ano de 2018, o hospital registrou aproximadamente mil atendimentos aos indígenas ^14^. Apesar da maioria dos profissionais serem naturais do estado, com mais de cinco anos de tempo de serviço e atenderem indígenas rotineiramente, nota-se o desconhecimento acerca dos povos e suas especificidades.

A invisibilidade dessa população ou a negação de suas especificidades é marcante na região; poucos são os sul-mato-grossenses que reconhecem a diversidade étnica ou que estão cientes da gravidade do cenário de violação de direitos enfrentado pelos indígenas. O racismo potencializado pelo contexto violento da disputa pela terra também influencia as práticas de saúde ^20^. Ademais, os cursos de graduação da área da saúde apresentam uma limitação ao não contemplar o conteúdo da antropologia e da etnologia, tendo como foco principal o biologicismo e a medicalização dos sujeitos ^19^ ^,^ ^21^. Ao desconhecer a identidade étnica como uma categoria importante na saúde, ignora-se os determinantes sociais e as iniquidades étnico-raciais que afetam o acesso aos serviços de saúde e a qualidade de vida ^22^.

Destaca-se que a Residência Multiprofissional em Saúde com ênfase em saúde indígena do HU-UFGD é única no Brasil e tem potencial para produzir transformações, favorecendo discussões acerca da interculturalidade e da antropologia da saúde entre residentes e profissionais no cotidiano do trabalho. Entretanto, até o momento da pesquisa, não houve projetos de educação permanente ou capacitações que agregassem as especificidades socioculturais e o atendimento diferenciado às populações indígenas.

Langdon ^23^ sugere que os profissionais de saúde devem estar atentos ao contexto sociocultural da população atendida e devem conhecer e respeitar os saberes indígenas para alcançar maior efetividade nas ações. No entanto, na prática, as condições e singularidades indígenas pouco são respeitadas pelos profissionais. Ribeiro et al. ^10^ analisaram os discursos de enfermeiros da CASAI do Mato Grosso do Sul e apontam indícios de etnocentrismo e negação das culturas indígenas. De modo geral, os profissionais não foram capazes de reconhecer as especificidades e saberes indígenas incluídas em universos distintos das concepções biomédicas já estabelecidas. O estudo destaca que tal situação é potencializada devido à burocratização do trabalho, à estrutura organizacional do serviço e às poucas ações de atividades de educação permanente.

A organização dos serviços de saúde e o modelo biomédico favorecem relações assimétricas entre profissionais e indígenas, estabelecendo prejuízos ao ideal de “atenção diferenciada” preconizado pelas políticas indigenistas. Ademais, a lógica estabelecida, ao desconsiderar outras formas de cuidado, reproduz estereótipos e discursos que justificam as falhas e as dificuldades do atual modelo de gestão ^24^.

### Articulação de saberes

A pesquisa aponta para uma baixa articulação entre diferentes formas de atenção à saúde quando a maior parte dos profissionais afirma que nunca presenciou algum cuidado tradicional dentro do hospital. As formas de atenção à saúde dos Kaiowá e Guarani articulam dimensões sociais, ambientais e espirituais e seu reconhecimento pelos serviços de saúde emerge como demanda política e como parte da reprodução biossocial do grupo ^25^ ^,^ ^26^.

A melhoria na qualidade da assistência à saúde, a solicitação de intérpretes e o respeito às práticas tradicionais são reivindicações dos Kaiowá e Guarani. O relatório produzido anualmente pelas mulheres na Kuñangue Aty Guasu (Grande Assembleia das Mulheres Guarani Kaiowá) cita violências obstétricas, perda do espaço e reconhecimento das parteiras indígenas, bem como a falta de articulação da biomedicina com a medicina tradicional ^27^.

É possível inferir que a instituição hospitalar ainda é percebida pelas indígenas como hostil aos cuidados tradicionais; que profissionais não reconhecem tais práticas, e/ou que os usuários as façam escondidas, com receio de represálias. Cruz & Guimarães ^12^ mencionam que durante a transferência da aldeia para cidade, os indígenas fazem uso de suas práticas terapêuticas; no entanto, por receio de serem proibidos, realizam-nas de maneira oculta.

No cenário hospitalar, essa articulação entre saberes parece ainda mais desafiadora, pois se caracteriza como um espaço por excelência do modelo biomédico, tecnicista e burocrático. Soma-se a isso a crescente medicalização de inúmeros aspectos da vida e a tendência do modelo médico hegemônico para ignorar outras formas de cuidado e de minimizar os determinantes sociais em saúde.

A articulação entre saberes biomédicos e indígenas é condição fundamental para a efetivação das políticas de saúde indígena/indigenista, para a garantia da integralidade e da atenção diferenciada, conforme prevê a PNASPI ^3^ ^,^ ^28^ ^,^ ^29^. No entanto, existe uma enorme distância entre o que é declarado e formalizado nas políticas de saúde e normatizações e o que ocorre no cotidiano dos serviços e no fluxo dos usuários ^30^.

Considera-se que os participantes declaram sentir, em algum nível, dificuldades nos encontros interétnicos. Tais achados também foram verificados em outros estudos ^5^ ^,^ ^9^. Santos et al. ^31^ avaliaram a comunicação entre usuários indígenas hospitalizados e a equipe de enfermagem, evidenciando as principais dificuldades que envolvem a falta de conhecimento, a habilidade insuficiente em assistir pessoas de outras culturas e a comunicação. Marinelli et al. ^32^, em relação à assistência à população indígenas no Maranhão e as percepções dos enfermeiros da equipe de saúde indígena, encontraram dificuldades sobre a falta de capacitação para o trabalho, a barreira linguística, as barreiras geográficas, a não aceitação do profissional pela comunidade e outras condições de trabalho não satisfatórias. Silva et al. ^5^ citam como dificuldades a questão linguística, as condições de trabalho e as questões culturais. Assim, é fundamental que profissionais sejam qualificados e participem de processos de educação permanente para ofertar um atendimento de qualidade e sensível a diferenças culturais.

É compreensível que residentes tenham uma visão mais positiva acerca da articulação com a medicina tradicional e da aproximação com aspectos étnico-culturais, já que estão em processo de aprendizado na pós-graduação, encontram-se em espaços de discussão, disciplinas, campos de prática no DSEI e a temática indígena permeia os espaços coletivos. Apesar de estarem incluídos na mesma categoria neste estudo, as Residências Médicas pouco compartilham das mesmas discussões que os programas multi e uniprofissionais. Como um hospital de ensino, há desafios na criação de espaços formais para o diálogo entre diferentes programas de residências e entre diferentes profissões.

Destaca-se que, apesar das diferenças significativas entre as categorias profissionais acerca da aproximação com o contexto indígena, as respostas variaram entre a mediana 9 e 10, indicando que profissionais consideram importante a aproximação com as comunidades indígenas, a fim de facilitar o diálogo e melhorar a assistência. Esse é um ponto crucial, pois a comunicação em saúde é um desafio no contexto intercultural.

Guimarães et al. ^33^ analisaram um projeto de extensão realizado na CASAI do Distrito Federal, no qual ocorreram rodas de conversa e oficinas para o compartilhamento de experiências pelos grupos étnicos atendidos. O projeto potencializou a subversão às práticas disciplinadoras e controladoras que comumente são vivenciadas nessas instituições de saúde. Além disso, também possibilitou nova perspectiva à formação dos estudantes e aos funcionários da CASAI.

### Percepções sobre o trabalho em equipe na assistência aos usuários indígenas

A diferença significativa entre profissionais e residentes sobre a equipe conseguir trabalhar de maneira harmonizada pode ser considerada por diversas nuances. Residentes passam de maneira temporária pelos setores e não fazem oficialmente parte da equipe. Parece razoável dizer que eles podem ter uma visão mais crítica sobre tal atuação, além de terem espaços formais para discussão de casos e reflexão sobre o cotidiano de trabalho.

Baquião et al. ^34^, em um estudo sobre a percepção de residentes multiprofissionais sobre o trabalho, encontraram que esses enfrentam dificuldades e desafios. Entre os principais problemas, foi identificado a própria formação acadêmica como obstáculo, além de problemas no diálogo com os profissionais de medicina.

É válido observar que em todos os setores hospitalares, as equipes médicas têm uma sala privativa disponível, compartilhada apenas com residentes médicos e internos de medicina, onde há espaço para estudo, discussão de caso, descanso e convivência. É possível que os médicos considerem o diálogo e a interação entre si mais positiva devido aos espaços privilegiados. Ademais, todas as decisões a respeito de usuários internados passam pela aprovação deles, que necessitam ser informados de qualquer procedimento não rotineiro.

É indiscutível que o cuidado multi e interdisciplinar acarreta inúmeros benefícios para os itinerários terapêuticos dos usuários indígenas e não-indígenas. No entanto, tal caminho ainda necessita de grandes avanços como discussão de casos, troca de informações e opiniões de especialistas ^35^. Os programas de Residência Multiprofissional em Saúde têm criado cada vez mais iniciativas que valorizem a educação inter e multidisciplinar, nos contextos da pós-graduação *lato sensu*, e, apesar dos desafios, exercem papel fundamental na produção de novas abordagens em saúde. No entanto, é necessário que esse caminho se inicie ainda no âmbito da graduação. Além disso, é fundamental a busca de relações mais horizontais e menos fragmentadas estabelecidas pelos profissionais com as comunidades.

Anjos Filho & Souza ^36^, em um estudo com profissionais de saúde, encontraram como obstáculos a carência de ações de educação permanente, a existência de dificuldades pessoais quanto ao compartilhamento de conhecimentos e a excessiva demanda de trabalho, sendo fundamental a criação de ferramentas de educação permanente, sobretudo em contexto hospitalar.

### A articulação da equipe hospitalar com a rede de cuidado em saúde indígena

Entre as ações que contemplam um atendimento adequado está a articulação em rede. A forma de organização entre o SasiSUS e as pactuações de referência e contrarreferência enfrentam desafios. A fragmentação dessa articulação dificulta a execução das políticas que buscam garantir a integralidade da assistência à saúde indígena. A gestão federal e a territorialização dos DSEI são especificidades que impactam na articulação federativa, refletindo diretamente na execução das políticas de saúde na rede SUS e impactando na prática diária dos profissionais de saúde ^37^.

Um estudo no DSEI de Cuiabá, Mato Grosso, encontrou baixa articulação entre os níveis de atenção, além de problemas logísticos de apoio e o predomínio de um modelo de serviços focado em ações especializadas e de alto custo. Verificou, ainda, a falta de articulação e de espaços de participação indígena e controle social ^38^.

Apesar dos avanços no reconhecimento ao direito à saúde das populações indígenas, é necessário aperfeiçoar as legislações e o comprometimento entre gestores das diferentes esferas de governo, a fim de garantir a integralidade da assistência.

## Considerações finais

Considerando o HU-UFGD como referência à população indígena, a atenção diferenciada preconizada pela PNASPI, ainda é um grande desafio. Os resultados evidenciam a centralidade do modelo biomédico, o desconhecimento por parte dos profissionais sobre o contexto sociocultural da população indígena atendida, além da desvalorização de seus saberes e práticas. Entre os achados, observou-se algumas diferenças entre residentes e profissionais e ainda entre categorias profissionais, com destaque para a categoria médica. Além disso, a maioria relatou sentir, em algum nível, dificuldades no atendimento à população indígena.

Os resultados contribuem para ampliar o debate acerca do tema, ainda pouco explorado, e para o aperfeiçoamento e a efetivação da equidade, da humanização e da atenção diferenciada, previstas nas políticas de saúde, apontando direcionamentos sobretudo no que tange à educação permanente dos profissionais de saúde.

Esses achados podem promover discussões sobre as políticas de saúde indígena nos diversos níveis de atenção e da gestão, bem como na construção de novas abordagens para melhorar o atendimento durante sua permanência hospitalar.

Também é preciso reconhecer como desafio a necessidade de ampliar a formação em saúde, caminhando no sentido de substituir o modelo segmentado e reducionista, por uma visão mais respeitosa e abrangente, que considere os diferentes saberes.
